# 2D constraint modifies packing behaviour: a halobenzene monolayer with X_3_ halogen-bonding motif

**DOI:** 10.1080/00268976.2021.1900940

**Published:** 2021-03-22

**Authors:** Jonathan A. Davidson, Stephen J. Jenkins, Fabrice Gorrec, Stuart M. Clarke

**Affiliations:** a Department of Chemistry, University of Cambridge, Cambridge, UK; b MRC Laboratory of Molecular Biology, Cambridge, UK; c BP Institute, University of Cambridge, Cambridge, UK

**Keywords:** Monolayer, physisorption, diffraction, DFT, halogen bonding, triiodotrifluorobenzene

## Abstract

Using a combination of X-ray diffraction and simulation techniques, we are able to identify a crystalline monolayer of 1,3,5-triiodotrifluorobenzene formed on graphite. The monolayer is found to exhibit an incommensurate hexagonal unit cell with a lattice parameter of 9.28(7) Å, exhibiting a trigonal arrangement of iodine atoms not found in the bulk structure. DFT simulations have been performed exhibiting close agreement with the experimental structure. Importantly these simulations can be used to compare the strength of the intermolecular interactions both with and without Van der Waals corrections. Thus it is possible to estimate that halogen bonding consists of approximately half the total interaction energy. This demonstrates that despite the presence of strong directional non-covalent bonding, dispersion interactions account for a very significant proportion of the total energy.

**Figure:**
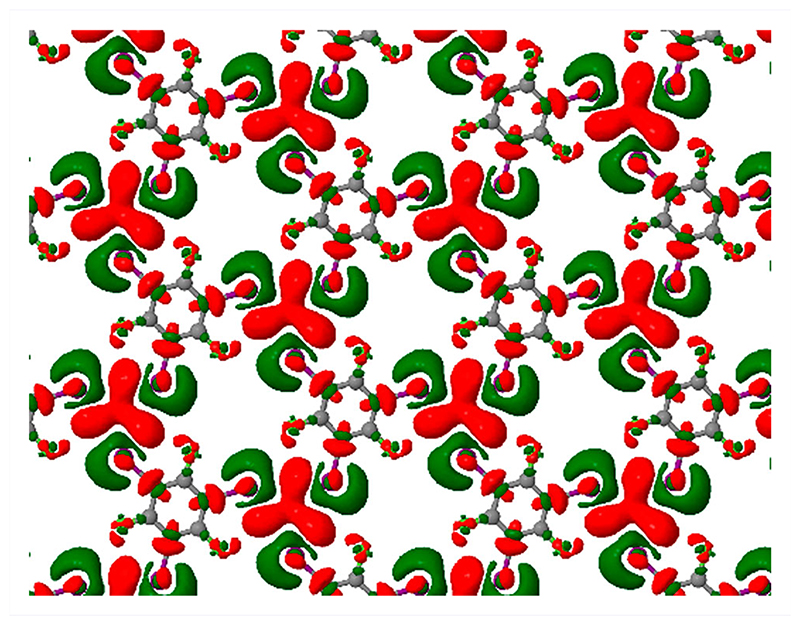

## 1 Dedication

We are honoured to dedicate this contribution to the memory of Prof. Dr. Gerhard Findenegg, whose work has been an inspiration to many within our field. Indeed, it was his careful and beautiful measurements, particularly those on the physisorbed monolayers formed by alkanes, fatty acids and alcohols, that encouraged one of us (SMC) to engage in this most fascinating topic and to explore the in-plane diffraction methods employed in this contribution. The present work also illustrates how the field has radiated from the hydrogen-bonded systems reported by Findenegg, to encompass other non-covalent interactions such as the halogen-bonded systems addressed here.

## 2 Introduction

Physical adsorption of molecules has been studied in a range of contexts by many authors. Gerhard Findenegg had a particular interest in the liquid–solid interface, performing many ingenious experiments using physical rules and rationalisation of gravimetric and volumetric changes to deduce the existence of solid monolayers of alkanes [1], carboxylic acids [2] and krypton [3] on graphite. Further imaginative and diligent experimentation helped uncover the thermodynamic properties of these monolayers [4,5].

Since then, a number of methods have been used to confirm the structures predicted. Alongside scanning tunnelling microscopy studies [6,7], a number have also been performed utilising X-ray and neutron diffraction. This has now been done for a range of physisorbed systems in their increasing complexity of intermolecular interactions: purely van der Waals e.g. alkanes [8–10] and fluoroalkanes [11,12]; hydrogen-bonded species such as alcohols [13], carboxylic acids [14] and amides [15,16]; and dipolar molecules such as halomethanes [11,17–19]. More recently, other halogen-containing species have been investigated [20,21], which has led to the observation of the first 2D halogen bonded monolayers [22], with several further studies addressing assembly by halogen bonding in monolayers [23–25].

These systems have become of interest due to the growing development of supramolecular chemistry at surfaces. Early examples of supramolecular systems at surfaces generally utilised hydrogen bonds [26,27], but there has been growing appreciation of the utility of alternative intermolecular interactions [28]. Halogen bonds provide a strong and directional counterpart to the hydrogen bond [29], involving different functional groups and hinting at the eventual development of systems assembled through an orthogonal mix of hydrogen- and halogen-bonding components.

**Figure 1:**
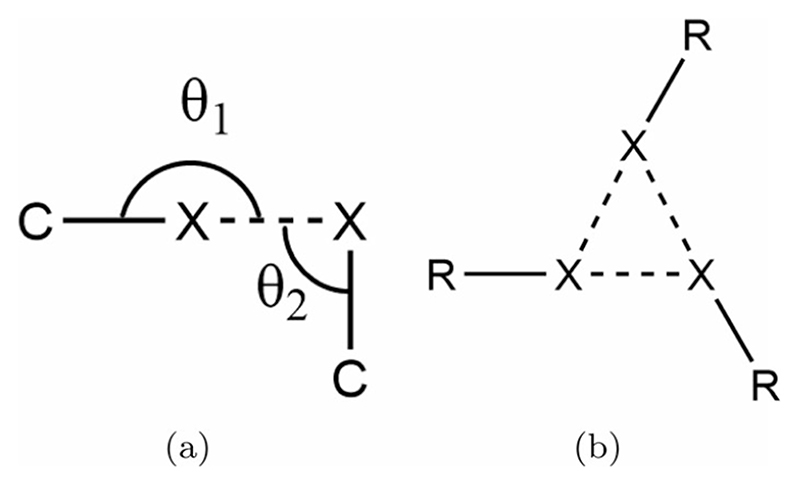
(a) Schematic of the key parameters *θ*_1_ and *θ*_2_ used to characterise halogen–halogen contacts. If *θ*_1_ ≈ *θ*_2_ the contact is type I and not considered halogen bonded. Type II contacts typically exhibit *θ*_1_ ≥ 150° and *θ*_2_ ≤ 120°. For an ideal halogen bond *θ*_1_ = 180° and *θ*_2_ = 90°. (b) Schematic of the X_3_ motif, which consists of three type II contacts in a trigonal geometry.

Not all close halogen–halogen contacts can be understood to be due to halogen bonds. When examining close contacts in bulk crystals, it is possible to divide these contacts into type I and type II contacts [30]. These are defined by the angles shown in Figure 1(a). Type I contacts exist where *θ*_1_ ≈ *θ*_2_ and are due to close-packing of large, polarisable atoms. Type II contacts ideally have *θ*_1_ = 180° and *θ*_2_ = 90°, and exist due to a region of positive charge on the terminal face of some halogen atoms, known as the σ-hole.

The phenomenology of the σ-hole can be rationalised in terms of either polarisation or orbital effects [31] and can be clearly observed through calculation of the electrostatic potential of the isolated halogen bond donor molecule [32]. The existence of such an area of positive charge, capable of interacting with nucleophiles, on atoms that would conventionally be described as negatively charged demonstrates how assignment of atomic point charges can be overly simplistic for the prediction of certain intermolecular interactions [33].

Whether calculated theoretically, or measured experimentally the common observation is that the magnitude of the σ-hole is greatest when X is least electronegative (I > Br > Cl) and bound to a highly electronegative atom/group. Due to these factors, when considering organic systems it is generally iodofluorocarbons that exhibit the strongest halogen bonds [34]. Previous work has indeed revealed robust halogen bonds between aromatic iodofluorocarbons and pyridyl groups on graphite surfaces [22,23].

A particularly intriguing halogen bonding motif is the so-called X_3_ synthon. This synthon (Figure 1 b) was first identified in the bulk crystal structure of trihalomesitylene molecules [35] and has since been recognised in several halogen bonding systems [29,36,37]. The motif consists of a trigonal arrangement as shown in Figure 1(b), with each halogen atom acting both as a halogen bonding donor and acceptor. Its significance is that it contributes a versatile threefold symmetric vertex for the construction of supramolecular scaffolds. Molecules exhibiting structural and chemical similarity to the trihalomesitylene compounds are therefore of significant interest, as they may also give rise to exploitable X_3_ motifs in their intermolecular interactions.

**Figure 2:**
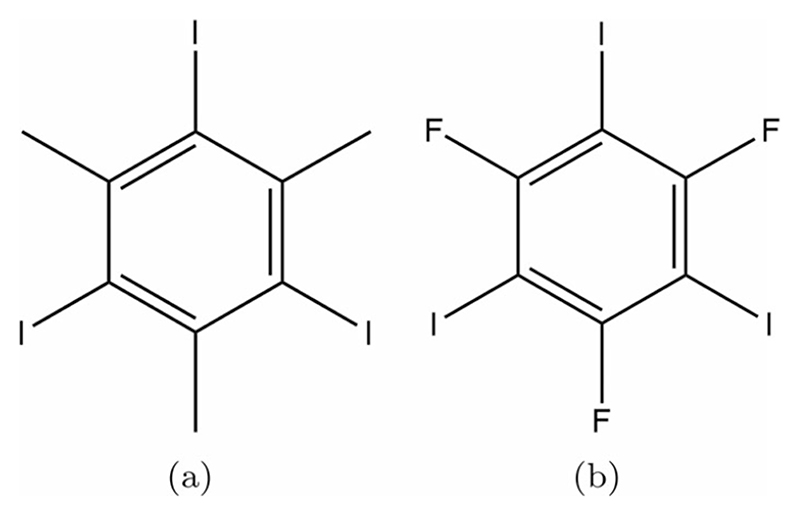
(a) Chemical structure of triiodomesitylene, the first species shown to exhibit the X_3_ halogen bonding motif. (b) Chemical structure of 1,3,5-triiodo-2,4,6-trifluorobenzene, the species considered in this work. Despite the structural similarity the fluorinated compound was found not to exhibit the X_3_ bonding motif in the bulk [38].

1,3,5-triiodo-2,4,6-trifluorobenzene (TITFB) is a fluorinated analogue to triiodomesitylene (Figure 2) and hence may show promise in the rational design of halogen bonding systems. It has already been utilised in several studies on halogen bonding in bulk co-crystals [39–41]. At the surface, it has been observed to interact with halogen-bond bases to form ordered structures under applied potential from an STM tip. Intriguingly, however, the structure of the single component TITFB monolayer has thus far resisted analysis [42,43]. Related molecules have been examined using low temperature STM on metal surfaces, including hexabromobenzene on gold (77 K) [44] and hexafluorobenzene on silver (5 K) [45]. To the present authors’ knowledge, however, no STM images of small perfluorinated aromatic molecules on graphite have previously been reported. It is possible that comparatively weak binding to the surface renders the layer prone to perturbation by the STM tip, or that the electronic properties of molecules of this type are unfavourable for high-resolution imaging.

In bulk, TITFB does not adopt the X_3_ structure (isomorphic to triiodomesitylene) that may have been expected. Instead, it has been observed to adopt a corrugated structure [38]. In contrast, lamellar X_3_ structures have been observed for 1,3,5-triiodo-2,4,6-trichloroben zene and 1,3,5-triiodo-2,4,6-tribromobenzene, so it is curious that the fluorinated molecule does not follow suit. This apparent anomaly was ascribed by Reddy et al. to the destabilising effect of large voids that would be left in the TITFB-based structure due to the significantly smaller size of fluorine atoms relative to bromine, chlorine or methyl groups [38].

In this work, we report the assembly of a TITFB monolayer that does indeed adopt an ordered structure on graphite based on the X3 motif. The structure has been characterised using X-ray diffraction and simulated using density functional theory (DFT), with remarkable agreement between the two techniques.

## 3 Methodology

### 3.1 Experimental

The experimental method adopted in this work has been detailed elsewhere [23]. A recompressed graphite foil known as ‘papyex’ is used as a substrate. This foil contains a large number of graphite crystallites that are highly aligned in the plane of the sheet, permitting manipulation of the diffraction geometry to optimise scattering from the in-plane monolayer peaks. Diffraction experiments were performed at the Laboratory of Molecular Biology (LMB) in Cambridge using a Rigaku FR-E+ superbright (rotating copper anode, 200-*μ*m beam) diffractometer. Peak intensity is obtained at 1.54179 Å, through the use of a graphite monochromator. A nitrogen cryostream (Oxford Cryostream) was used to cool the sample to 100 K. A MAR-DTB area detector was used with a detector-sample distance of 250 mm. This gave a maximum 2*θ* range of 34°. Calibration of the detector angles was performed using a papyex strip coated in silver behenate. Integration of the obtained powder rings onto a single radial dimension was performed using the fit2D software platform [46,47], and further analysis utilised a custom python script (patternNx) that accounts for the observed ‘sawtooth’ lineshape of 2D diffraction peaks [23,48].

To prepare the samples, papyex (BET surface area of 15.61 m^2^.g^−1^) was first outgassed under vacuum for 6 h at 673 K. It was then placed into a glass tube with a weighed amount of 1,3,5-triiodo-2,4,6-trifluorobenzene (Acros Organics, 97%) and sealed under vacuum. The tubes were then heated to 473 K and allowed to slowly anneal before the dosed papyex was recovered. Dosing was performed such that coverage was submonolayer (0.8 ML) based on an estimate of the molecular area.

### 3.2 Computational

In order to gain insight into the strength and nature of intermolecular bonding in this system, we performed first-principles DFT calculations, making use of the CASTEP computer code [49]. Previous experience with similar systems [23,24,50] has confirmed that interactions between halogen-bonded overlayers and an underlying graphite substrate are often sufficiently slight that the latter can safely be omitted from consideration within such calculations. Accordingly, we modelled the TITFB monolayer as a raft of molecules, applying periodic boundary conditions consistent with a supercell of length 12 Å (perpendicular to the raft) and lateral dimensions conforming initially to a hexagonal lattice constant of 9.30 Å (chosen close, but not identical, to the experimentally determined value). The lateral lattice constant was permitted to relax, along with the internal structure of the TITFB molecule replicated within each supercell, in response to the calculated forces, subject only to the constraint that the lattice was to remain of simple hexagonal type. Convergence of the evolving geometry was gauged with respect to an energy tolerance of 10^−5^ eV, a force tolerance of 0.02 eV.Å^−1^, and a stress tolerance of 0.01 GPa.

Consistent with periodic boundary conditions, the Kohn–Sham wavefunctions of the system were expanded in a plane-wave basis set, up to a kinetic energy cutoff at 600 eV, while the Brillouin zone was sampled over a 2 × 2 × 1 Monkhorst-Pack mesh [51]. Electron–ion interactions were included through the use of ultrasoft pseudopotentials [52] and the exchange-correlation interaction was represented by the Perdew–Burke–Ernzerhof (PBE) functional [53]. Sensitivity of the results to Van der Waals (VdW) interactions, which are not satisfactorily described within the standard DFT approach, was tested by comparing the effects of two semi-empirical correction schemes, namely the Tkatchenko–Scheffler (TS) scheme [54] and Grimme’s D2 scheme [55]. Intermolecular interaction energies were estimated by comparing total energies computed in the fully relaxed supercells (for each scheme) against energies computed for molecules relaxed within supercells artificially fixed at twice the equilibrium lateral lattice constant (in which circumstances the interactions between neighbouring molecules are expected to be negligible). Calculations performed in such ‘expanded’ cells (but with frozen local molecular geometries) were also the source of reference electron density distributions, which could be subtracted from the electron density obtained at the equilibrium lateral lattice constant to provide ‘electron density difference’ maps that reveal spatial information on the formation of intermolecular bonds.

## 4 Results

### 4.1 Experimental data

**Figure 3:**
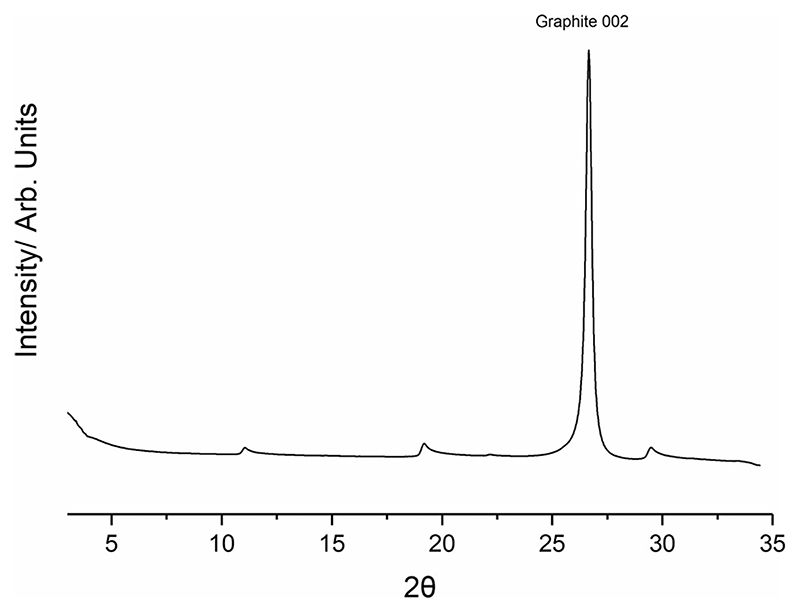
Collected diffractogram of papyex dosed with TITFB, the Graphite 002 peak is labelled. Below 5° small-angle scattering is significant. In addition to the bulk graphite peak several additional ”sawtooth” peaks of much lower intensity can be seen.

The collected diffractogram for graphite dosed with TITFB is presented in Figure 3. There is a large symmetric peak at 2*θ* = 27° consistent with the 002 reflection of the graphite substrate. In addition to this, there are several asymmetric ‘sawtooth’ type peaks evident only in the dosed graphite diffractogram. The most significant of these peaks occur at 2*θ* = 11°, 19° and 30°. The ‘sawtooth’ lineshape is diagnostic of 2D layers and strongly indicates formation of a monolayer. The long trailing edge is associated with the presence of Bragg rods in the plane-perpendicular direction, indicating a lack of periodicity perpendicular to that plane and ruling out an origin in any three-dimensional structure.

To isolate the diffraction peaks belonging solely to the monolayer, the diffraction pattern of clean papyex can be subtracted from the raw diffractogram. The subtraction close to the graphite 002 peak is imperfect, making it difficult to interpret any features, so this region has been ignored in the fitting. The process used to analyse the reliable peaks has been explained elsewhere.[56] In brief, the peak positions are used to determine the 2D unit mesh, with high symmetry solutions being preferred to help constrain the fitting procedure. The positions of molecules within this cell are then adjusted to match the experimental peak intensities. Due to the comparatively few reflections observed, the molecule is treated as a rigid body to limit the total degrees of freedom. The reported bulk crystal structure [38] is used to obtain an initial model of the rigid body.

The peak positions are well indexed to a hexagonal unit mesh with p3 symmetry (*a* = *b* = 9.28(7) Å, *γ* = 60°). This cell is not immediately commensurate to the underlying graphite lattice *a* = 2.461 Å and so indicates that intermolecular interactions are dominant over molecule–substrate interactions. The area of this cell matches well with that of a single TITFB molecule lying flat, and this interpretation is further supported by the threefold symmetry of the unit mesh (since the rotational symmetries of the molecule and of the unit cell can match only if the molecule lies flat on the surface). This greatly constrains the position of the TITFB molecule, and indeed treating TITFB as a rigid body leaves rotation within the plane of the substrate as the only remaining degree of freedom. We express this degree of the freedom by means of a rotation angle, ω, here defined relative to the molecular orientation in which iodine atoms from adjacent TITFB molecules point directly along primitive lattice vectors.

**Figure 4:**
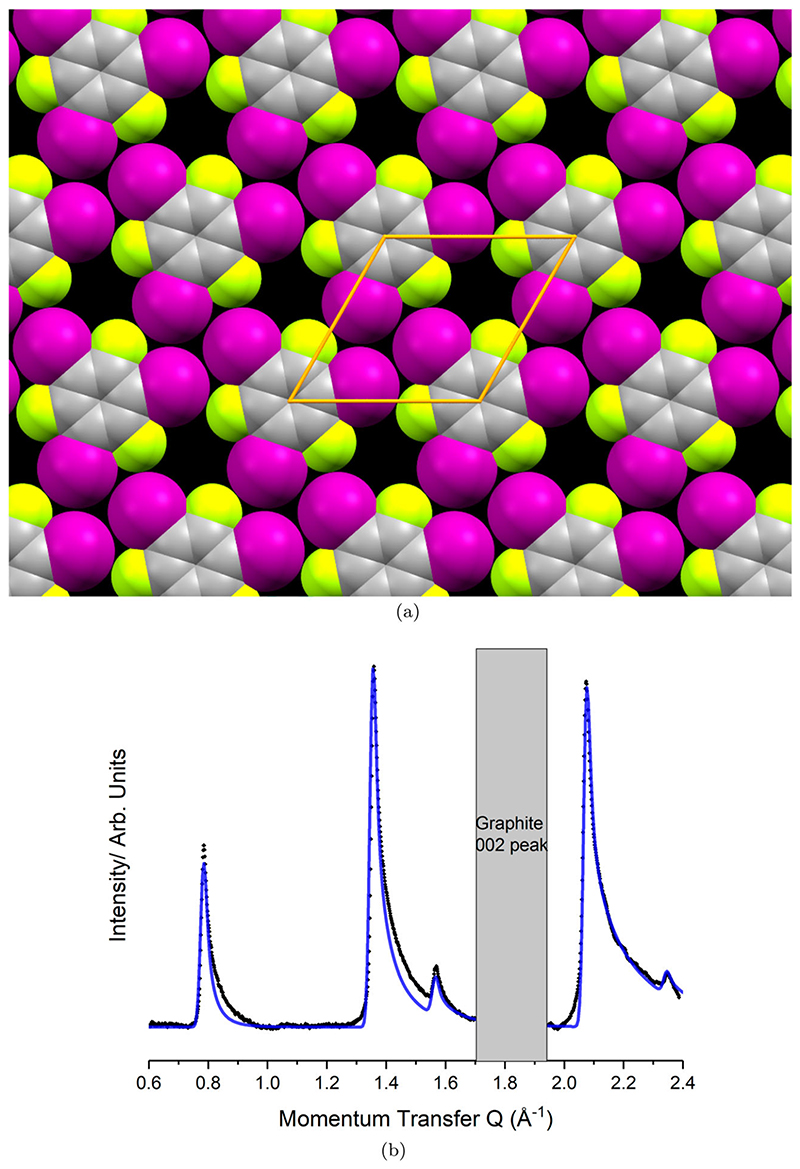
Comparison between the collected (black) and modelled (blue) diffraction pattern for the TITFB monolayer structure shown in (a), (a) Optimised experimental structure for the TITFB monolayer. The unit mesh is hexagonal with lattice parameters *α* = 9.28(7) Å *γ* = 60°. (b) Background subtraction of the monolayer pattern (black) compared to the modelled pattern (blue).

The best fit to the data is achieved with ω = 11.5°, leading to the monolayer structure depicted in Figure 4(a). It is evident that the iodine atoms adopt the distinctive X_3_ motif, and that there are gaps in the layer due to the small size of the fluorine atoms. Each C–I bond points very nearly directly towards an iodine atom in a neighbouring molecule, yielding values of *θ*_1_ = 163.2° and *θ*_2_ = 103.2° and suggesting a Type II (halogen bond) interaction. In contrast, the C–I bonds point quite wide of the fluorine atoms on neighbouring molecules, yielding *θ*_1_ = 148.2° and *θ*_2_ = 148.0° and suggesting a Type I interaction (not a halogen bond). Figure 4(b) compares the predicted diffraction pattern for this model to the experimentally collected data after substrate subtraction. An excellent match can be seen between the experimental data (black) and the modelled data (blue). Rotation of just a few degrees in either direction dramatically worsens the peak intensity fit. The slight underestimation of intensity in the first peak is likely due to the absence of Debye–Waller factors in this model, which are ignored so as to constrain the fitting. As TITFB is a fairly rigid molecule, the magnitude of these temperature effects would be expected to be minimal.

The structure displayed in Figure 4 can be compared to planes of the bulk lamellar structures reported for 1,3,5-triiodoiiodomesitylene[35] and 1,3,5-tridiodotri chlorobenzene[38]. Table 1 compares a few key parameters for these systems alongside the DFT structures reported below. The distance between adjacent molecules is slightly smaller for the TITFB monolayer, but the I… I separations are slightly larger (4.1 Å vs. 3.9 Å). This is due to the relatively small C–I… I *θ*_1_ angle in the TITFB system, with the iodine atoms pointing less directly towards each other so as to minimise the empty space around the small fluorine atoms.

### 4.2 Simulation

The optimisation of the DFT model structure in the absence of VdW corrections yielded a lateral lattice constant of 9.40 Å, which is 1.2% greater than the experimental value. Inclusion of the TS correction reduced this to 9.31 Å (0.2% higher than experiment) while the D2 correction led to a value of 9.26 Å (0.3% smaller than experiment). Non-rigidity of the simulated molecule precludes reporting any single angle comparable to the experimentally determined rotation (*ω* = 11.5°)butthecarbonring was rotated by 15.1° in the absence of VdW corrections, by 14.1° upon inclusion of the TS corrections, and by 13.9° when the D2 correction was employed. In all cases, it was confirmed that 0° and 30° rotation angles were less stable, by at least 0.07 and 0.73 eV per molecule respectively.

The calculated geometries all feature an X_3_ motif similar to that deduced from the experimental data. In the absence of VdW corrections, the C–I… I angles are *θ*_1_ = 171.4° and *θ*_2_ = 111.4°, characteristic of Type II (halogen bond) interactions, while the C–I… F angles are *θ*_1_ = 140.2° and *θ*_2_ = 139.9°, indicative of Type I (not halogen bond) interactions. Including the TS correction, we find *θ*_1_ = 168.4° and *θ*_2_ = 108.4° for the C–I… I angles, with *θ*_1_ = 142.0° and *θ*_2_ = 141.5° for the C–I… F angles, while use of the D2 correction yields *θ*_1_ = 168.1° and *θ*_2_ = 108.0° for the C–I… I angles, with *θ*_1_ = 142.1° and *θ*_2_ = 141.9° for the C–I… F angles. Calculated I… I distances (3.90 –3.92Å) are a little shorter than those found in our experiments, but still consistent with typical halogen bond lengths in comparable systems (e.g. triiodomesitylene or triiodotrichlorobenzene). Although our calculated I… F distances (3.57–3.71 Å) are even shorter, the small size of fluorine implies that any interaction involving dissimilar halogen atoms should make only a somewhat secondary contribution to the overall intermolecular bonding.

From the energetic standpoint, comparison against calculations conducted in supercells constrained at twice the equilibrium lateral lattice constant nevertheless provides clear evidence for substantial intermolecular interactions. In the absence of VdW corrections, the energy cost incurred in artificially doubling the lattice constant amounted to 0.16 eV per molecule, while the corresponding figures for the TS- and D2-corrected calculations were 0.34 and 0.44 eV per molecule respectively. These results suggest that halogen-bonding (present even without VdW corrections) amounts to between one-third to one half of the total intermolecular interaction, the remaining contribution being dispersive in nature.

**Figure 5:**
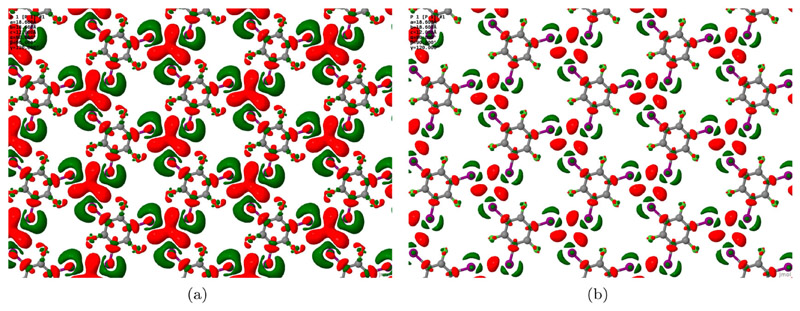
Maps of electron density accumulation (red) and depletion (green) for the condensed TITFB monolayer, relative to the electron density of isolated molecules constrained to the same local geometries but with doubled lateral lattice constant. Isosurface thresholds are set at(a) ±2 × 10^−3^ electrons.Å^−3^ and (b) ±4 × 10^−3^ electrons.Å^−3^.

Further insight into the nature of intermolecular bonding was sought by examining the electron density difference that arises when halving the (artificially doubled) lateral lattice constant. Figure 5 shows the results for the D2-corrected case, but a similar qualitative picture would doubtless pertain to the other two simulation types. Isosurfaces reveal that electron density accumulates predominantly in the region central to the X_3_ motif, balanced by depletion of electron density in the vicinity of the iodine atoms. Such a pattern is entirely consistent with our previous examination of electron density difference plots in halogen-bonded systems, where depletion of electron density close to the acceptor and donor atoms was accompanied by accumulation of electron density in the mid-bond region [23,24].

## 5 Conclusion

Using a combination of experimental and theoretical techniques, the halobenzene TITFB has been shown to form a crystalline monolayer on graphite. The monolayer exhibits the X3 bonding motif, with iodine atoms from adjacent molecules meeting at angles typical for a type II halogen bonded contact. By contrast the iodine–fluorine interactions between adjacent molecules are type I (dispersive) in nature. That TITFB on graphite forms the X_3_ motif, in contrast to its behaviour in bulk shows how constraint to two dimensions can radically modify intermolecular binding patterns relative to those seen in three dimensions.

## Figures and Tables

**Table 1:** Key geometric parameters for several relevant systems. *θ*_1_ and *θ*_2_ are defined in Figure 1 (a).

System	a	C–I… I			C–I… F		
		*θ*_1_	*θ*_2_	*d*(*I* …*I*)	*θ*_1_	*θ*_2_	*d*(*I* …*F*)
TITFB expt	9.29	163.2	103.2	4.08	148.2	148.0	3.48
TITFB (No VdW)	9.40	171.4	111.4	3.92	140.2	139.9	3.71
TITFB (TS VdW)	9.31	168.4	108.4	3.90	142.0	141.5	3.64
TITFB (D2 VdW)	9.26	168.1	108.0	3.90	142.1	141.9	3.57
Bulk triiodomesitylene from [35]	9.59	170.7	119.4	3.90			
Bulk triiodotrichlorobenzene from [38]	9.44	169.5	115.6	3.83			
